# Closing the Gap: Integrating Science and Practice in Psychotherapy

**DOI:** 10.1192/j.eurpsy.2025.2156

**Published:** 2025-08-26

**Authors:** N. Schwarzbach, M. Pijnenborg, R. Hoekstra, A. Poppe, T. Bouman

**Affiliations:** 1 University of Groningen, Groningen, Netherlands

## Abstract

**Introduction:**

The science-to-practice gap in psychotherapy is a prominent topic of discussion that hinders the seamless integration of research findings into clinical settings. This divide seems, among others, to stem from conflicting views on the practical relevance of evidence-based mental health (EBMH).

**Objectives:**

This study aims to provide a comprehensive overview of the existing narratives that define the science-to-practice gap, develop an inclusive definition that reflects the complexities of this issue, and identify the factors influencing and strategies for mitigating this gap.

**Methods:**

We conducted a systematic literature review with a qualitative, thematic synthesis approach including 131 articles. Themes were identified and synthesized to outline the science-to-practice gap. Additionally, we included a historical analysis to examine how the prevalence of certain codes and themes has evolved over time, reflecting shifts in the academic and clinical landscape.

**Results:**

Based on our findings, we refined the definition of the science-to-practice gap, capturing its multifaceted nature. Key themes influencing this gap include the educational background of psychotherapists, orientation towards specific psychotherapeutic schools, and personal inclinations of psychotherapists. Contextual factors such as institutional support and incentives for employing EBMH were also found to be positive influences. However, critiques regarding the rigidity of research methodologies and their applicability to diverse clinical scenarios were prevalent, with observable variances in thematic emphasis over the decades. Strategies identified for bridging the gap emphasized increased dialogue and collaboration between researchers and practitioners.

**Image 1:**

**Figure:**
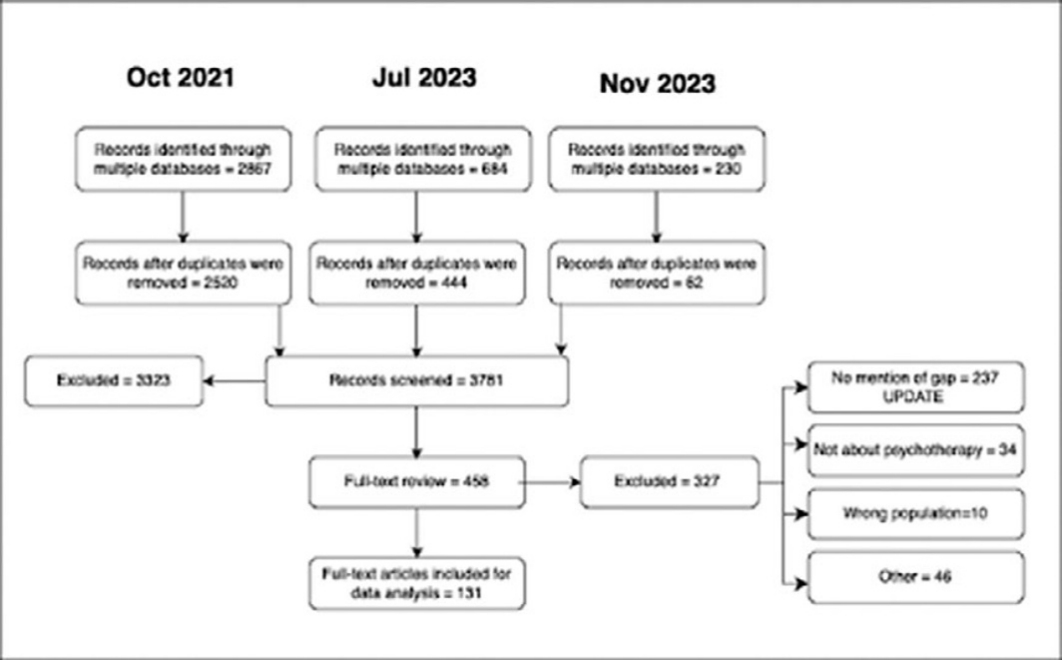

**Image 2:**

**Figure:**
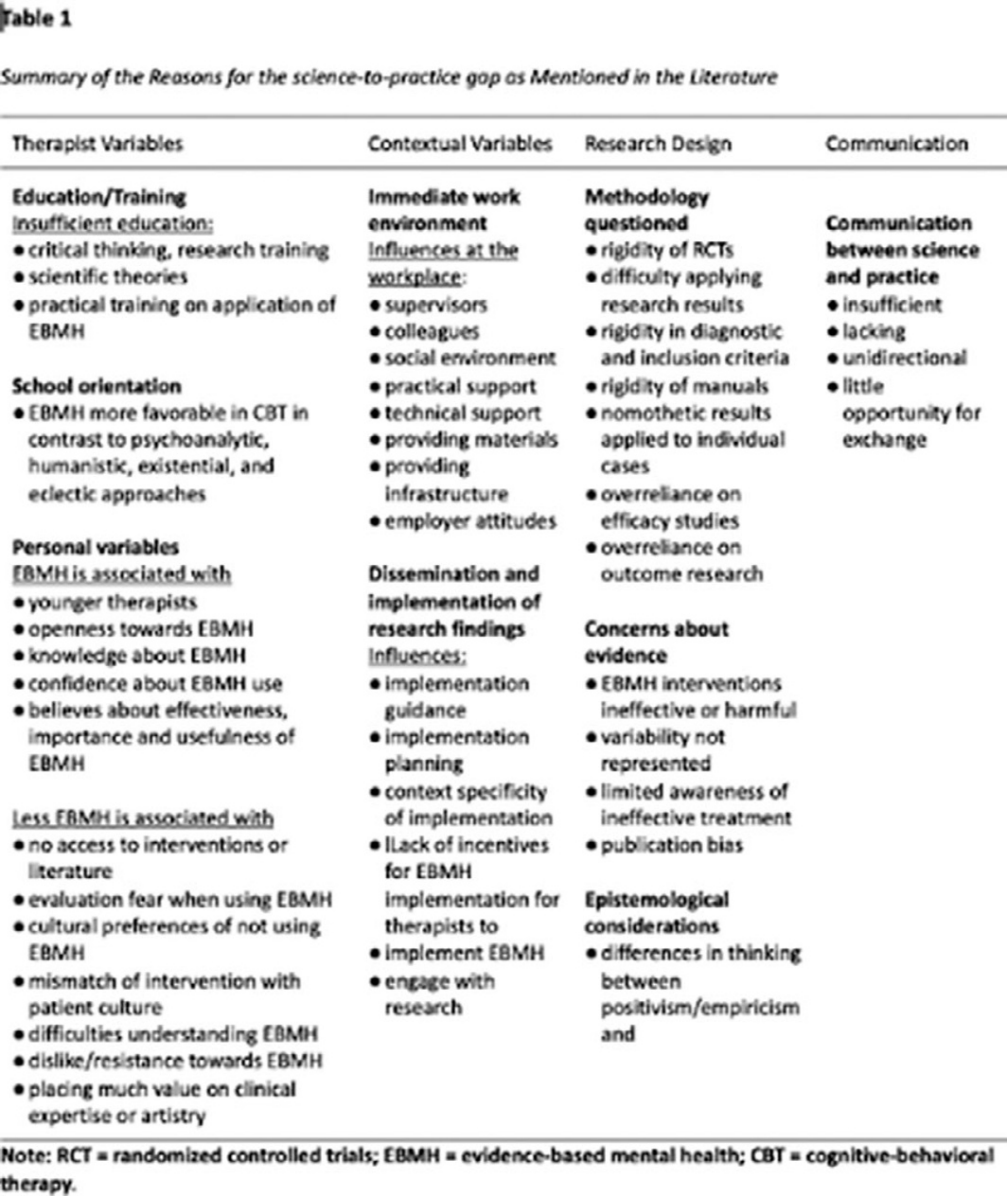

**Image 3:**

**Figure:**
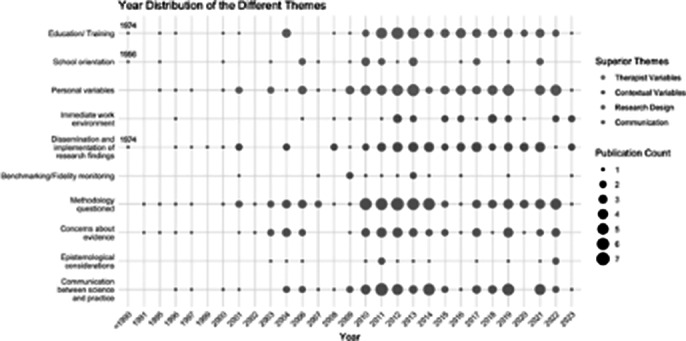

**Conclusions:**

This work sets the stage for future research that should prioritize the clinical perspective on evidence usefulness, broaden the research focus beyond intervention efficacy, and validate diverse methodologies. By proposing practice-focused research guidelines and emphasizing the need for robust dialogue between science and practice, we aim to enhance the applicability of research findings in clinical settings. Ultimately, our findings advocate for policies that facilitate the exchange of ideas and experiences, aiming to bridge the gap between scientific evidence and psychotherapeutic practice effectively.

**Disclosure of Interest:**

None Declared

